# Analysis of trastuzumab and chemotherapy in advanced breast cancer after the failure of at least one earlier combination: An observational study

**DOI:** 10.1186/1471-2407-6-63

**Published:** 2006-03-15

**Authors:** Rupert Bartsch, Catharina Wenzel, Dagmar Hussian, Ursula Pluschnig, Ursula Sevelda, Wolfgang Koestler, Gabriela Altorjai, Gottfried J Locker, Robert Mader, Christoph C Zielinski, Guenther G Steger

**Affiliations:** 1 Department of Internal Medicine I, Division of Oncology, Medical University of Vienna, Vienna, Austria

## Abstract

**Background:**

Combining trastuzumab and chemotherapy is standard in her2/*neu *overexpressing advanced breast cancer. It is not established however, whether trastuzumab treatment should continue after the failure of one earlier combination. In this trial, we report our experience with continued treatment beyond disease progression.

**Methods:**

Fifty-four patients, median age 46 years, range 25–73 years, were included. We analysed for time to tumour progression (TTP) for first, second and beyond second line treatment, response rates and overall survival.

**Results:**

Median time of observation was 24 months, range 7–51. Response rates for first line treatment were 7.4% complete remission (CR), 35.2% partial remissions (PR), 42.6% stable disease > 6 months (SD) and 14.8% of patients experienced disease progression despite treatment (PD). Corresponding numbers for second line were 3.7% CR, 22.2% PR, 42.6% SD and 31.5% PD; numbers for treatment beyond second line (60 therapies, 33 pts 3^rd ^line, 18 pts 4^th ^line, 6 pts 5^th ^line, 2 pts 6^th ^line and 1 patient 7^th ^line) were 1.7% CR, 28.3% PR, 28.3% SD and 41.6% PD respectively. Median TTP was 6 months (m) in the first line setting, and also 6 m for second line and beyond second line. An asymptomatic drop of left ventricular ejection fraction below 50% was observed in one patient. No case of symptomatic congestive heart failure was observed.

**Conclusion:**

The data presented clearly strengthen evidence that patients do profit from continued trastuzumab treatment. The fact that TTP did not decrease significantly from first line to beyond second line treatment is especially noteworthy. Still, randomized trials are warranted.

## Background

Breast cancer remains the main cause of cancer morbidity and mortality in women in most countries all over the world [1,2]. While localised disease is potentially curable, even in stage I and II disease, 30 % of patients can be expected to experience a relapse [3]. Especially at risk for cancer recurrence are patients of young age, nodal positive tumours and individuals with aggressive tumour phenotypes, defined as high or intermediate grade, endocrine receptor negative and/or her2/*neu *positive [4,5]. When metastatic disease develops, appropriate therapeutic strategies are necessary to lengthen the patient's survival while not further reducing her quality of life.

In her2/*neu *positive tumours, a combination of chemotherapy and trastuzumab has proven to produce superior response rates and a longer time to progression than chemotherapy alone [6-10]. Trastuzumab is a monoclonal humanized antibody targeting the epidermal growth factor receptor 2 (her2/*neu*), resulting in an anti-tumour activity whose exact mechanism of action is not yet fully understood. Antibody dependent cytotoxicity (ADCC) is part of this mechanism, but also the blocking of post-receptor pathways and the inhibition of homo- and hetero-dimerization are thought to play a crucial role [11-13]. A benefit however can only be found in tumours with her2/*neu *3+ over-expression in immunohistochemistry or in cells with her2/*neu *gene amplification, which is usually analysed by FISH (fluorescence in situ hybridisation) [14]. Paclitaxel plus trastuzumab was the first combination regimen established [15]. While in vitro studies were able to demonstrate an additive anti-tumour effect of this combination, other substances (vinorelbine, docetaxel, and cisplatin) showed a synergistic effect [16]. In vivo, it was possible to demonstrate, that vinorelbine plus trastuzumab regimens are not only superior to paclitaxel containing regimens in terms of toxicity [17], but also in terms of response and survival [9,10,16,18]. A recent study found docetaxel plus trastuzumab highly superior to docetaxel monotherapy as first line palliative treatment with little additional toxicity [19].

Today, a first line palliative combination of chemotherapy and trastuzumab can already be deemed standard. Still, it is not established whether or not patients do achieve a benefit from continuing trastuzumab treatment combined with a different chemotherapeutic agent after the failure of one earlier combination, as the way a resistance to trastuzumab develops is not yet understood entirely. Because of limited resources, also pharmacoeconomic aspects must be taken in account.

At our centre, patients were routinely treated with trastuzumab also in the second line and beyond second line setting. Individuals were observed prospectively, and we are reporting our experiences with trastuzumab treatment after the failure of at least one earlier trastuzumab containing therapy regimen.

## Methods

All data were collected at the Department of Internal Medicine I, Division of Oncology at the Medical University of Vienna, Vienna, Austria. Treatment was performed in accordance with the ethical regulations of the Medical University of Vienna.

### Patients

Fifty-four consecutive patients were included into to this trial after progressing on first line trastuzumab based treatment, and were followed prospectively. Prospective follow up of the first patient started in May 2002. The retrospective starting point of the study (i.e. the date the first patient received the first cycle of trastuzumab based 1^st ^line therapy) was June 2001. All patients were suffering from histological confirmed her2/*neu *(HERcepTest+++/FISH positive) positive advanced breast cancer and were treated with at least two palliative lines of trastuzumab containing therapy regimens. For staging evaluations, CT-scan of the chest and the abdomen, mammography, echocardiography, and gynaecologic examination were mandatory at baseline.

### Treatment plan and patient evaluation

All treatment was administered in the outpatient setting. Patients received trastuzumab in a dose of 8 mg/kg body weight loading dose on the first day of treatment, followed by 6 mg/kg body weight every three weeks [20]. Re-evaluation of patients' tumour status was performed with CT-scan of the chest and the abdomen with additional work up if indicated every three cycles of therapy. Echocardiography was repeated at irregular intervals, longest every 6 months. Complete response (CR) was defined as the disappearance of all measurable lesions for a minimum of eight weeks. Partial response (PR) was defined as 25% or more reduction in sum of products of the greatest diameters of measurable lesions, no increase of lesion size and no new lesions. Stable disease (SD) was defined as less than 25% decrease and less than 25% increase without the appearance of new lesions. Progressive disease (PD) was defined as greater than 25% increase in tumour size or the appearance of new lesions.

### Statistical analysis

Time to progression (TTP) was defined as the interval from the first day of application of a new therapy line until tumour progression. Data was analysed as of August 2005. TTP was estimated using the Kaplan-Meier product-limit method [21]. To test the difference between TTP curves, the log-rank test was used. *p *values less than 0.05 were considered to indicate statistical significance. Toxicity was evaluated according to the WHO criteria and was recorded per patient as the worst episode that occurred on a certain therapy. Echocardiography data were recorded, and development of left ventricular function during treatment was reported.

## Results

### Patient characteristics

Fifty-four patients (53 female/1 male) suffering from her2/neu positive metastatic breast cancer were included in this evaluation. Median age was 46 years, range 25–73 years. Table 1 lists the characteristics of all patients included. 89.8% had invasive ductal carcinoma and 10.2% were suffering from invasive lobular carcinoma. Twenty-three patients had positive estrogens receptors and fifteen also a positive progesterone receptor. Thirty-one patients were treated with adjuvant chemotherapy, with nineteen patients receiving anthracyclin based regimens and thirteen taxane containing therapies. Adjuvant endocrine therapy was administered in 15 patients (14 patients received tamoxifen and one anastrozole). Twelve patients had metastases at the time of primary diagnosis. Median time to development of metastases in the other 42 patients was 23 months, range 2 – 210 months. Six patients had only visceral metastases, ten metastases in the bones and/or the soft tissue, and the remaining 38 patients had metastases of both risk groups. Median number of metastatic sites was 3, range 1–7 sites. In 14 individuals, a trastuzumab containing combination was not the first palliative line of treatment. Those patients had received one earlier line of palliative therapy with aromatase inhibitors without trastuzumab.

Most common combination partners were: vinorelbine (n = 55), docetaxel (n = 28), capecitabine (n = 28), gemcitabine (n = 25), platinum derivatives (n = 13), others (n = 28).

### Response and survival data

**Figure 1:**
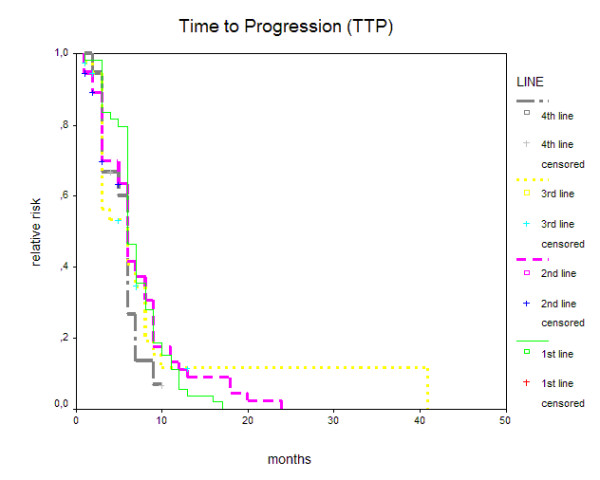
Time to progression (months) for 1^st^, 2^nd^, 3^rd^, and 4^th ^line trastuzumab based combination therapy.

All fifty-four patients are evaluable for response and toxicity. Median time of observation (all trastuzumab containing treatment lines) was 24 months, range 7 – 51 months. Median time of observation in the first line setting was 6 months (range 1 – 17 months), and corresponding numbers were median 6 months (range 1 – 24 months) for second line, 5 months (range 1 – 41 months) for third line, 5.5 months (range 3 – 10 months) for fourth line, 4.5 months (range 1 – 12) for fifth line, 7 months (range 6 – 8 months) for sixth line, and 3 months for seventh line respectively. Response rates for first line combination treatment were 7.4% complete remission (CR), 35.2% partial remissions (PR), 42.6% stable disease (SD) and 14.8% of patients experienced disease progression despite treatment (PD). Corresponding numbers for second line were 3.7% CR, 22.2% PR, 42.6% SD and 31.5% PD; numbers for treatment beyond second line (60 therapies, 33 pts 3^rd ^line, 18 pts 4^th ^line, 6 pts 5^th ^line, 2 pts 6^th ^line and 1 patient 7^th ^line) were 1.7% CR, 28.3% PR, 28.3% SD and 41.4% PD respectively. Two patients showed response to a sixth line treatment. Forty-six patients (85.2%) experienced a clinical benefit (CR + PR + SD≥6 months) from first line treatment, 68.5% from second line, and 35/60 treatments (58.3%) beyond second line. TTP was 6 months (m) (range 1–17 m, 95% CI 5.40 – 6.60) in the first line setting, 6 m (1–24 m, 95% CI 5.36 – 6.64) in the second line and also 6 m (1–41 m, 95% CI 5.32 – 6.68) beyond second line. Log rank test revealed no significant difference between the groups. TTP for 3^rd ^line was 6 m (range 1–41 m, 95% CI 2.79–9.21), and 4^th ^line 6 m (range 2–10+ m, 95% CI 5.34–6.66) respectively (Figure 1). TTP for 5^th ^line treatment was 6 months (range 2–12 m, 95% CI 2.74–9.26) and actual progression time was median 6 m. No Kaplan-Maier estimations are available for 6th and 7th line treatment, as only one patient has yet progressed on 6^th ^line therapy (after 8 months), and the only patient receiving a 7th line treatment progressed after 3 months. Median survival was not reached after 24 months. Two patients, who experienced disease progression on first line treatment, responded with PR to second line therapy. Of three patients with PD in second line (from a total of sixteen), one showed a PR and four SD during later lines of therapy. Two patients were lost to follow up at the time of analysis and were therefore not evaluable for overall survival.

### Toxicity

All side effects are shown in Table 2. Trastuzumab based therapy was well tolerated and we observed no case of treatment related death. There was also no case of symptomatic congestive heart failure. In one patient, left ventricular ejection fraction dropped below 50% without symptoms, therefore necessitating a discontinuation of therapy. Still, this decrease was fully reversible, and output rate normalised over the next 12 months.

Other toxicities observed were caused by the chemotherapy agents used as combination partners. A WHO grade IV neutropenia was observed in 5 patients, with no other grade IV toxicity reported. WHO grade III toxicities included stomatitis (2 patients), nausea (1 patient), neutropenia (14 patients), thrombocytopenia (2 patients), hand-foot-syndrome (3 patients) and anaemia (6 patients).

## Discussion

While there is some evidence from different other, mostly retrospective, trials reporting a benefit from continued trastuzumab treatment beyond disease progression with a changed chemotherapy regimen only [22-24], a discontinuation after disease progression is still standard of care. Though the here presented study is limited by the relatively small number of patients included, we are clearly able to strengthen existing evidence that there is a benefit for at least some patients.

The reported decline in response rates from 42.6% in first line treatment to 30% in beyond second line compares to the expected drop of response rates with every further line of chemotherapy or endocrine therapy in palliative treatment. Stable disease and objective response combined, clinical benefit rates were 85.2% in first line, 68.5% in second line and 58.3% in beyond second line. As some other groups, we believe this to be the more significant parameter in judging the efficacy of palliative treatment, as a stabilisation of the disease without excessive toxicity often appears more important than objective remission [25,26]. The high clinical benefit rate of nearly 60% even beyond second line must be seen as a clear sign of a benefit that most patients gain from treatment continuation. It is also obvious from our here presented data that this benefit is not paid for by excess toxicity, as apart from a possible drop in cardiac output rate, no other major toxicity linked to trastuzumab was observed. This was especially important to us, as some patients included in this trial were treated with trastuzumab for more than 3 years. Notably, we did not find a case of symptomatic congestive heart failure. All other toxicities were well within the range expected from the different chemotherapy regimens. Still, in one patient, treatment had to be discontinued because of a drop in ejection fraction below 50%. So while trastuzumab based regimens are well tolerated also in medium and long term treatment, a monitoring of cardiac function remains essential. Echocardiography only in the case of symptoms appears not to be sufficient.

Of special interest is the fact, that we did not find a statistical significant difference in TTP between first line, second line and beyond second line trastuzumab based combinations. Although we rather expected to observe a shortening of time to disease progression with every further line of therapy, as this is the usual development in the metastatic setting, we believe this to be another sign of a potential benefit from continued combination treatment. TTP data from the different treatment lines (median 6 months in 1^st ^line, 2^nd ^line and beyond 2^nd ^line) compare favourably to the data reported in the pivotal trastuzumab trial with median 6.9 months in combination with paclitaxel as first line therapy [27]. As median overall survival was not yet reached, we believe that our survival data will be well beyond the median 25.4 months presented in the pivotal trial. These results are intriguing and somewhat unexpected, but still the necessity for larger, especially randomised, trials remains.

A big problem lies in the fact, that the mechanism of resistance to trastuzumab is not yet fully understood. If tumour cells are able to switch the main growth pathway from the her2/*neu *receptor to the EGFR or other signal transduction pathways, a continued treatment would not necessarily lead to a benefit. On the other hand, if no complete resistance develops, a discontinuation of trastuzumab might cause a massive overshoot in tumour growth, in which case only combination partners should be switched [28,29]. With limited resources available, also economic aspects must be taken on account.

## Conclusion

In conclusion, we are definitely able to strengthen evidence that her2/*neu *positive patients do profit from continued trastuzumab therapy, with a clinical benefit rate of 68.5% in the second line. We are to our best knowledge the first group reporting a similar TTP in 1^st ^line, 2^nd ^line and beyond 2^nd ^line treatment, making our data especially noteworthy. Further, this is among the first studies reporting a beneficial role of trastuzumab combination even beyond second line. Albeit the here presented data, randomised trials are still warranted to make a final conclusion regarding treatment continuation possible.

## Declaration of competing interests

The author(s) declare that they have no competing interests.

## Authors' contributions

RB contributed the idea for this trial, participated in the design and drafted the manuscript, CW participated in the statistical analysis and helped drafting the manuscript, DH participated in the design of the study, UP and US both participated in the collection of patient data, WK helped in the design and coordination of the study, GA recorded patient data, GL revised the manuscript critically, RM assisted in the statistical analysis, CZ and GS were important in the design of the study, the coordination and helped to draft the manuscript.

All authors have read and approved the final manuscript.

## Pre-publication history

The pre-publication history for this paper can be accessed here:



## Figures and Tables

**Table 1:** Patient characteristics

Characteristics	Patients
Entered	n = 54
Age median (years) (range)	46 years (range 25 – 73 y)
Estrogen receptor/progesterone receptor positive	23/15
HERcepTest+++/FISH+	51/3
primary metastatic disease	12 (22.2%)
Metastatic sites median (range)	3 (range 1–7 sites)
Lung	22
Liver	29
Bones	30
Lymph nodes	22
Soft tissue	36
Skin	11
Brain	11
Others	3
More than one metastatic site	48 (88.8%)
Adjuvant chemotherapy	31 (57.4%)
Adjuvant docetaxel	13
Adjuvant anthracyclines	19
Adjuvant endocrine therapy	15 (27.7%)
Palliative endocrine therapy	20 (37%)
1^st ^line combination	n = 54 pts
Vinorelbine	35
Capecitabine	2
Gemcitabine	0
Docetaxel	12
Platinum derivatives	0
Others	5
2^nd ^line combination	n = 54 pts
Vinorelbine	15
Capecitabine	11
Gemcitabine	9
Docetaxel	11
Platinum derivatives	0
Others	8
Beyond 2^nd ^line combination	n = 60 therapies
Vinorelbine	6
Capecitabine	14
Gemcitabine	14
Docetaxel	4
Platinum derivatives	11
Others	11

**Table 2:** Toxicities (n = 54 patients). (toxicities shown as worst episode per therapy)

Toxicity	WHO grade			
	I	II	III	IV
Nausea/Vomiting	1 (1.8%)	13 (24.1%)	1 (1.9%)	-
Neutropenia	-	4 (7.4%)	14 (25.9%)	5 (9.3%)
Thrombocytopenia	-	5 (9.3%)	2 (3.7%)	-
Anaemia	18 (33.3%)	7 (13%)	6 (11.1%)	-
Diarrhoea	-	4 (7.4%)	-	-
Stomatitis	-	2 (3.7%)	2 (3.7%)	-
Polyneuropathia	1 (1.8%)	2 (3.7%)	-	-
Hand-Foot-Syndrom	3 (5.6%)	10 (18.1%)	3 (5.6%)	-
